# Stress Distribution Pattern in Mandibular Overdenture Designs Supported by Three Dental Implants: A 3D Finite Element Analysis

**DOI:** 10.1002/cre2.70060

**Published:** 2025-03-02

**Authors:** Negin Aminianpour, Marzieh Alikhasi, Mostafa Shabanpour Kasari, Hashem Yousefi, Hakimeh Siadat

**Affiliations:** ^1^ Dental Research Center, Dentistry Research Institute Tehran University of Medical Sciences Tehran Iran; ^2^ Department of Prosthodontics, School of Dentistry Tehran University of Medical Sciences Tehran Iran; ^3^ Department of Mechanical Engineering Iran University of Science and Technology Tehran Iran; ^4^ Research and Development Unit Avita Dental Implant System, KFP‐Dental Tehran Iran; ^5^ Department of Biomedical Engineering Islamic Azad University, Tehran South Branch Tehran Iran

**Keywords:** computer‐aided design, dental implants, denture, finite element analysis, overlay, von Mises stress

## Abstract

**Objectives:**

The purpose of this study was to assess the implant‐supported overdenture stress distribution pattern in cancellous and cortical bone, dental implants, and prosthetic components, and its displacement by using finite element analysis (FEA).

**Materials and Methods:**

An edentulous model of the mandible was designed with three dental implants placed at the sites of canine teeth and the midline. Six groups were designed with isolated (ball and locator) and splinted (conventional bar and a CAD/CAM milled bar with cast and screw ball) attachments with and without a cantilever using SolidWorks 2022 software. The stress distribution pattern in the surrounding bone, implants, and prosthetic components (attachments, caps, housings, and screws) was evaluated following the application of 150 N force vertically and 105 N load with a 30° angle relative to the first molar site using Abaqus/CAE 2021 software. The implant‐supported overdenture displacement was also evaluated.

**Results:**

The ball attachment caused the highest stress in the attachments (363 and 896 MPa) and housings (375 and 1187 MPa) under vertical and oblique loadings, and cancellous bone (6 MPa under vertical loading). The pattern of stress distribution was variable following vertical and oblique loading in the cortical bone and dental implants in different groups. The locator attachment resulted in lower stress distribution in bone, implants, and prosthetic components. In splinted groups, the cantilever designs caused lower stress in bone, implants, and prosthetic components in comparison with designs without a cantilever. The conventional and milled bar did not show any mechanical difference.

**Conclusion:**

Considering the stress distribution patterns, the locator attachment is preferred to the ball isolated attachment, and the cantilever design is preferred among the splinted types. Isolated attachments were more effective in controlling the displacement. Also, conventional and milled bars did not show any superiority over each other.

## 1 Introduction

Implant‐supported mandibular overdentures are increasingly being used due to their improved stability, retention, function, and esthetics, promoting the quality of life of patients (Cicciù et al. 2015; Dimililer et al. 2018; Oda et al. 2017; Doundoulakis et al. 2003). Different types of attachments may be used for implant‐supported mandibular overdentures (Cicciù et al. 2015), which have been evaluated in several studies (Daas et al. 2008; Celik and Uludag 2007; Prakash et al. 2009; Barão et al. 2013). Many studies reported that the locator attachment caused lower stress distribution in the attachment, dental implants, and cancellus bone, compared with the ball and bar attachments (Geramy and Habibzadeh 2018; Jiang et al. 2019; Trang et al. 2022; El‐Anwar et al. 2015; Bhattacharjee et al. 2022).

The computer‐aided design and computer‐aided manufacturing (CAD‐CAM) technology can be used for the fabrication of overdenture attachments (Sa et al. 2021). Studies comparing the stress distribution and displacement patterns of conventional attachments with CAD‐CAM fabricated types have shown controversial results (Kim and Hong 2016; Kümbüloğlu et al. 2022; Shishesaz et al. 2016). Shishesaz et al. (2016) reported smaller displacement in isolated (ball) attachments compared with splinted attachments (bar‐ball and bar‐clip) in implant‐assisted mandibular overdentures supported by three dental implants. Idzior‐Haufa et al. (2021) reported higher displacement in the no‐cantilever design (bar) compared with the attachment with a cantilever (bar with CEKA in the distal part) in overdentures supported by two dental implants. Implant‐assisted mandibular overdentures may be supported by two to five dental implants (Scortecci et al. 2019). However, Bassi‐Bassi‐Junior et al. (2021), in a finite element study, reported that three‐implant‐assisted mandibular overdentures showed more satisfactory force dissipation than the four‐implant type.

The majority of the available relevant studies (Barão et al. 2013; Geramy and Habibzadeh 2018; Jiang et al. 2019; Trang et al. 2022; El‐Anwar et al. 2015; Bhattacharjee et al. 2022; Kim and Hong 2016; Kümbüloğlu et al. 2022; Shishesaz et al. 2016; Brandt et al. 2021) evaluated only a few types of attachments, and did not comprehensively assess this topic. Some reported lower stress in the isolated attachment (Barão et al. 2013; Trang et al. 2022; El‐Anwar et al. 2015; Shishesaz et al. 2016) and some reported lower stress in the splinted attachment (Geramy and Habibzadeh 2018; Jiang et al. 2019). Also, studies comparing milled bars with other attachments are limited in number (Kim and Hong 2016; Kümbüloğlu et al. 2022; Shishesaz et al. 2016). Some reported lower stress in the isolated attachment (Barão et al. 2013; Trang et al. 2022; El‐Anwar et al. 2015; Shishesaz et al. 2016) and some reported lower stress in the splinted attachment (Geramy and Habibzadeh 2018; Jiang et al. 2019). Thus, it is important to assess the effect of the aforementioned parameters on overdentures supported by three dental implants.

The purpose of this study was to assess the overdenture stress distribution pattern in cancellous and cortical bone, dental implants, and prosthetic components (attachments, caps, housings, and screws) and also its displacement by using finite element analysis (FEA). The null hypothesis of the study was that the attachment type and the direction of load application (vertical vs. oblique) would have no significant effect on the overdenture stress distribution pattern and displacement.

## 2 Materials and Methods

### 2.1 Preparation of the CAD File of the Model

An edentulous model of the mandible was designed, and its extracted CAD file was used for this FEA. SolidWorks 2022 software (Dassault Systems) was used to design the geometrical model of the mandible with two layers of cortical and cancellous bones. A cone‐beam computed tomography scan of the mandible of a 54‐year‐old man was used for this purpose after obtaining his consent. After extracting the CAD file of the mandible, the cancellous bone was designed using 1‐mm sections perpendicular to the mandibular cross‐section. The remaining area was a hollow shape with 2‐mm average thickness, which simulated the cortical bone. Soft tissue and mucosa were designed with 1–3‐mm thickness (Cruz et al. 2006).

### 2.2 Insertion of Dental Implants

Three implants (AVITA, Kousha Fan Pars Co., BLP3712) with 3.7 mm diameter and 12 mm length were also modeled. Bone‐level implants were inserted into bone with tight contact. Two implants were inserted at the sites of right and left canine teeth, and one implant was inserted at the midline, with a 15.5 mm distance from each other. For dental implant insertion, the base of the mandible and the mandibular canal was taken into account according to the cone‐beam computed tomography scan of the patients to select the most suitable size and position for dental implants.

### 2.3 Modeling of the Overdenture

Six groups were designed with isolated (ball and locator) and splinted (conventional bar and CAD/CAM milled bar) attachments with and without a cantilever using SolidWorks 2022 software. The six study groups are presented in Table 1. The splinted groups were designed in ExoCAD software (Exocad DentalCAD; Exocad GmbH) with the same contour, but the milled bar was made of titanium and the waxed‐up bar was made of a nickel–cobalt–chromium alloy. The geometrical models of LOC3 and Ball3 groups were designed according to the AVITA Morse‐Hex connection. AVITA BLP3712 has a 22° morse and a 2.5‐mm hexagonal diameter of the peripheral circle. A compatible ball abutment was used with 5 mm gingival height and 3.5 mm diameter similar to the locator abutments. Finally, the denture was designed and added to the model. A cap was considered for the locator and a ring was considered for the ball attachments.

**Table 1:** Properties and figures of all six study groups.

Groups	Properties
WAX2	A U‐shaped waxed bar with two balls, cast with a nickel–cobalt–chromium alloy.
WAX4	U‐shaped waxed bar, cast with a nickel–cobalt–chromium alloy with four balls, two of which were posterior to the last implants bilaterally (cantilever design).
Ball3	ball attachment (AVITA, Kousha Fan Pars Co., Iran) with 3.5 mm diameter and 5 mm gingival height.
CAD2	U‐shaped milled titanium alloy (Ti6Al4V) bar with two screw holes for balls. The balls were scanned and added to the milled bar.
CAD4	A U‐shaped milled titanium alloy (Ti6Al4V) bar with four screw holes for balls, two in the middle, and two posterior to the last implants bilaterally (cantilever design). The balls were scanned and added to the milled bar.
LOC3	BPLA3750 locator attachment (Kerator, KJ Meditech Co. Ltd, Korea) with 3.7 mm diameter and 5 mm gingival height.

### 2.4 Assembly and Exporting of the Models

The denture was connected to a splinted or isolated attachment with a metal housing and a female. Furthermore, after placement of the denture on the housing and female, it could easily move and rotate within a small range, allowing load distribution and preventing joint failure.

Finally, the geometrical CAD models were converted into STEP format by SolidWorks 2022 and the STEP files were subsequently exported to Abaqus/CAE 2021 (Dassault Systems Simulus Corp.).

### 2.5 Preparation of the Finite Element Model

**Figure 1:**
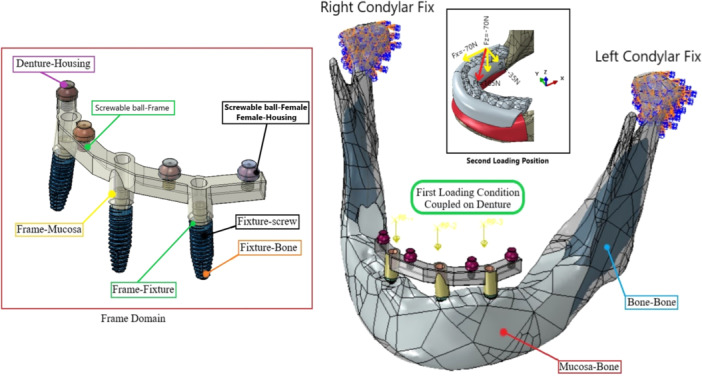
A tetrahedron mesh element with the C3D4 nodes was used in all models. This cross‐sectional view from the CAD2 group demonstrates the mesh density at the bone–implant contact.

All materials and structures were considered to be homogenous, isotropic, and linearly elastic. The implants were considered to have perfect (ideal) osseointegration (Cruz et al. 2003, 2009). Ties and bonds at the interfaces, including bone to bone, framework to implant, ball abutment/locator to implant, mucosa to bone, screw to implant, screw to framework, framework to mucosa, and housing to denture, are shown in Figure 1 (Topkaya and Solmaz 2015; Arat Bilhan et al. 2015). The remaining interactions, including ball to framework, female to housing, female to ball, denture to mucosa, ring to locator, and ring to ball abutment, were considered frictional (Daas et al. 2008; Spazzin et al. 2011).

Accordingly, the coefficient of friction was considered to be 0.35 for the framework–ball, 0.3 for the ring–ball, 0.3 for the cap–locator, 0.2 for the ball–female, 0.2 for the housing–female, and 0.01 for the denture–mucosa (Barão et al. 2013; Spazzin et al. 2011). All degrees of freedom of the condyles in the mandible were also limited (Figure 1). All material models were considered to be isotropic. Table 2 presents the physical properties (Young's modulus of elasticity and Poisson's ratio) of the overdenture components and bone.

**Table 2:** Elastic modulus and Poisson's ratio of the materials (Cruz et al. 2003, 2009; Topkaya and Solmaz 2015; Bilhan et al. 2015; Spazzin et al. 2011; Emami et al. 2019; Celebic et al. 2023).

Materials	Elastic modulus (MPa)	Poisson's ratio
Cortical bone	13,700	0.3
Cancellous bone	1370	0.3
Mucosa	1	0.37
Ti6Al4V	110,000	0.34
Nickel–Cobalt–Chromium	212,000	0.31
Rubber	5	0.3
Denture	4500	0.35

To simulate the loads applied to the denture in the oral cavity, a 150 N load was applied vertically and symmetrically to the first molar site (Figure 1) (Arat Bilhan et al. 2015; Spazzin et al. 2011). This load was applied in six steps progressively to achieve more static conditions. In the second loading cycle, a 105 N oblique load was applied to the first molar site with a 30° angle in the XZ plane (Barão et al. 2013; Spazzin et al. 2011).

**Figure 2:**
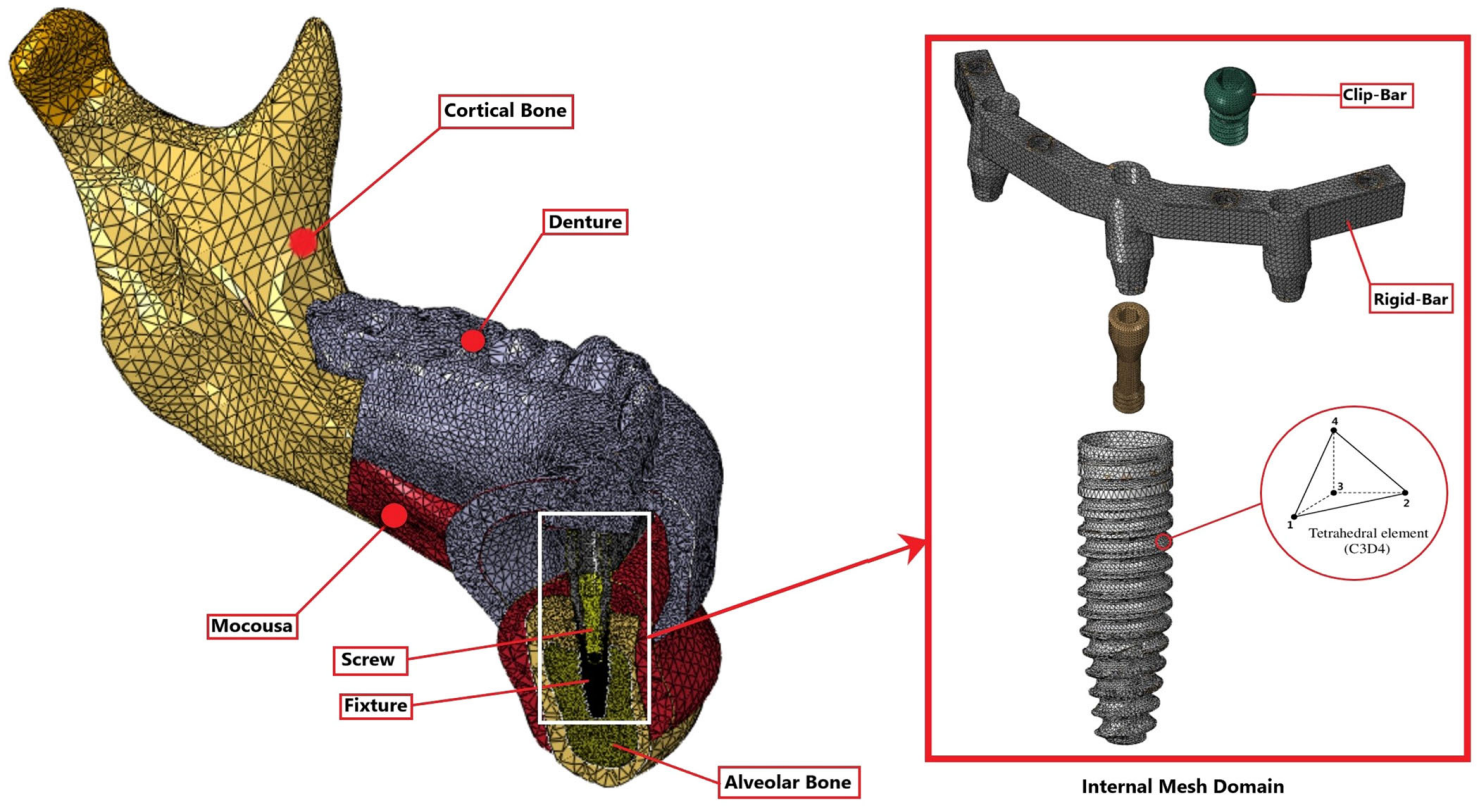
Interactions, boundary conditions, and loading types. (Left) Framework design with seven different interactions. According to CAD4, all of these interactions can be tied or frictional. The two condylar sections of the mandible were completely fixed in the right side. Vertical and oblique loading directions can be seen.

The geometrical models were then meshed using C3D4 linear tetrahedral elements in Abaqus/CAE 2021, which means that each element had three nodes. The number of solid items in each group is presented in Table 3. Sensitivity mesh analysis was subsequently performed for mathematical validation. A higher density meshing was considered for the connections, as in the bone–implant contact, denture connection with the housings, and attachment connection with the implants. Figure 2 shows the cross‐sectional view of a meshed model.

**Table 3:** Number of solid elements in each component of the six study groups.

Parts	WAX2	WAX4	CAD2	CAD4	LOC3	Ball3
Cortical bone	95,488	95,488	95,488	95,488	95,488	95,488
Cancellous bone	544,681	544,681	544,681	544,681	544,681	544,681
Mucosa	40,813	40,813	40,813	40,813	40,813	40,813
Implant	379,317	379,317	379,317	379,317	379,317	379,317
Screw	172,062	172,062	172,062	172,062	—	—
Rigid framework	87,607	81,818	—	—	—	—
Female–Ring–ball	33,312	79,204	33,288	79,204	28,692	44,382
Housing	15,226	34,292	15,174	34,292	15,636	40,902
Denture	178,383	183,154	188,593	183,154	230,577	214,528
Rigid framework + ball	—	—	125,897	220,799	—	—
Ball abutment	—	—	—	—	—	137,589
Locator	—	—	—	—	137,400	—
Total elements	1,546,889	1,591,028	1,595,313	1,749,810	1,472,604	1,497,700

For calculation of the von Mises stress values, the dental implants and prosthetic components were considered ductile. The von Mises stress was calculated using the following formula:

$$\sigma_{\mathrm{vm}}=\sqrt{{\sigma_{x}}^{2}+{\sigma_{y}}^{2}-\sigma_{x}\times \sigma_{y}+3\tau^{2}}$$

where *σ*_vm_ is the von Mises stress, *σ*_x_ and *σ*_y_ are the normal stresses in the *x* and *y* directions, respectively, and *τ* is the shear stress in the *x–y* plane. For the three‐dimensional analysis, the formula is extended to include a third direction and considers the shear stress in two more planes.

## 3 Results

**Figure 3:**
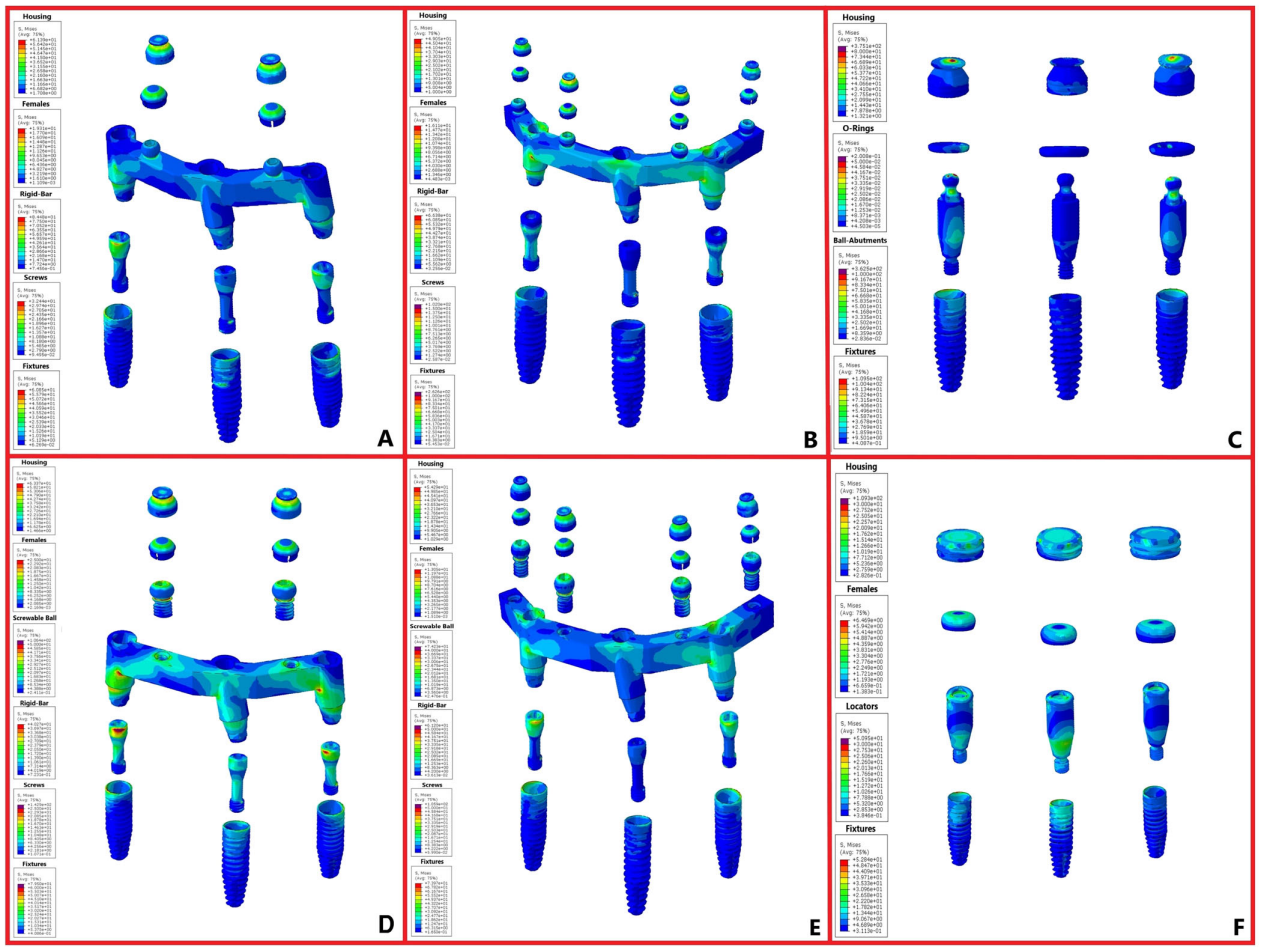
Stress distribution under vertical loading in the six study groups: (A) WAX2, (B) WAX4, (C) Ball3, (D) CAD2, (E) CAD4, and finally (F) LOC3. The color‐coded indicators and their spatial distributions highlight the critical points and areas of higher stress concentration.

**Figure 4:**
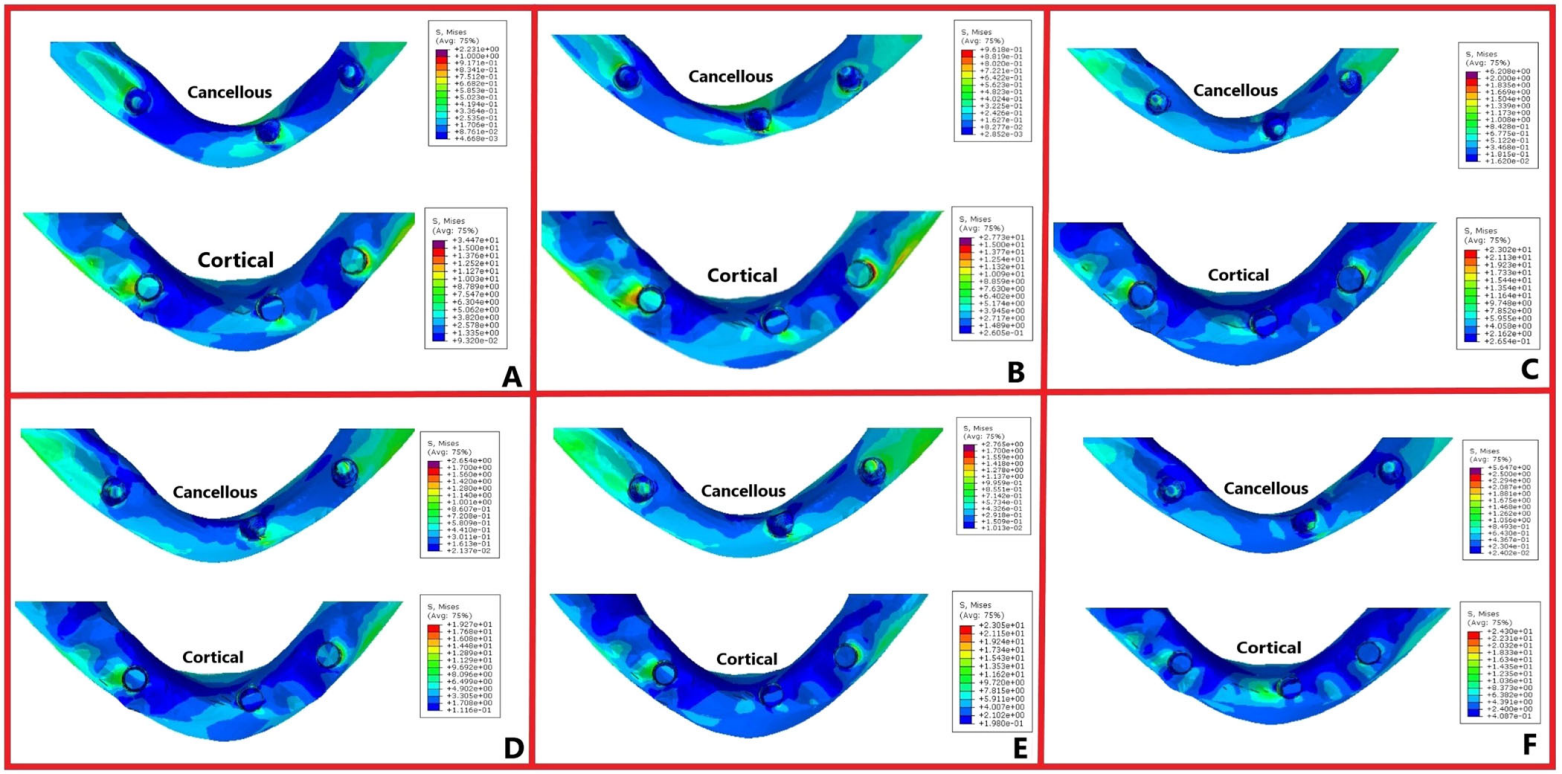
Stress distribution pattern under vertical loading of the cortical and cancellous bone in the six overdenture models of (A) WAX2, (B) WAX4, (C) Ball3, (D) CAD2, (E) CAD4, and (F) LOC3 in megapascals (MPa).

**Figure 5:**
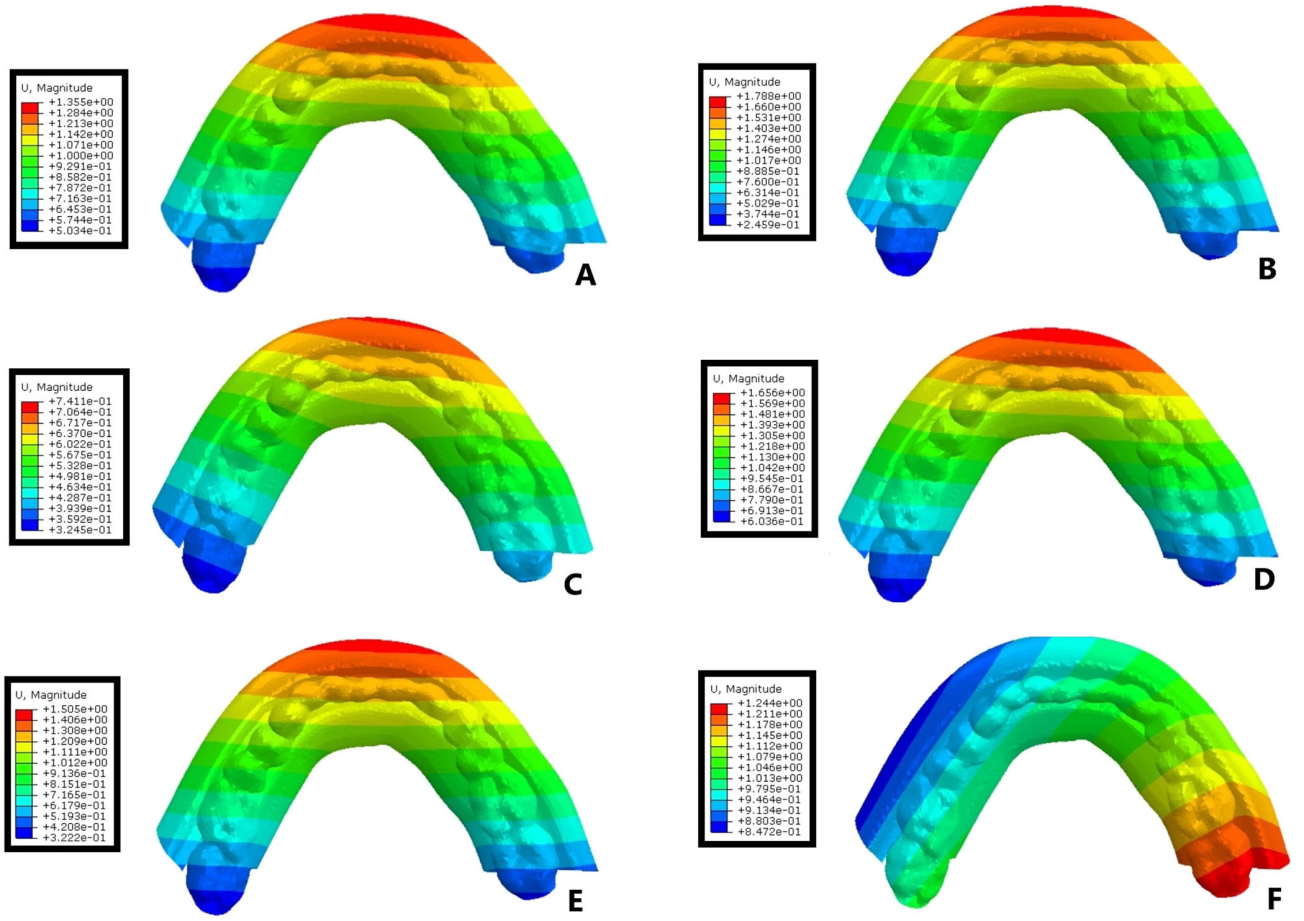
Denture displacement (mm) in the six overdenture models under vertical loading: (A) WAX2, (B) WAX4, (C) Ball3, (D) CAD2, (E) CAD4, and (F) LOC3.

**Figure 6:**
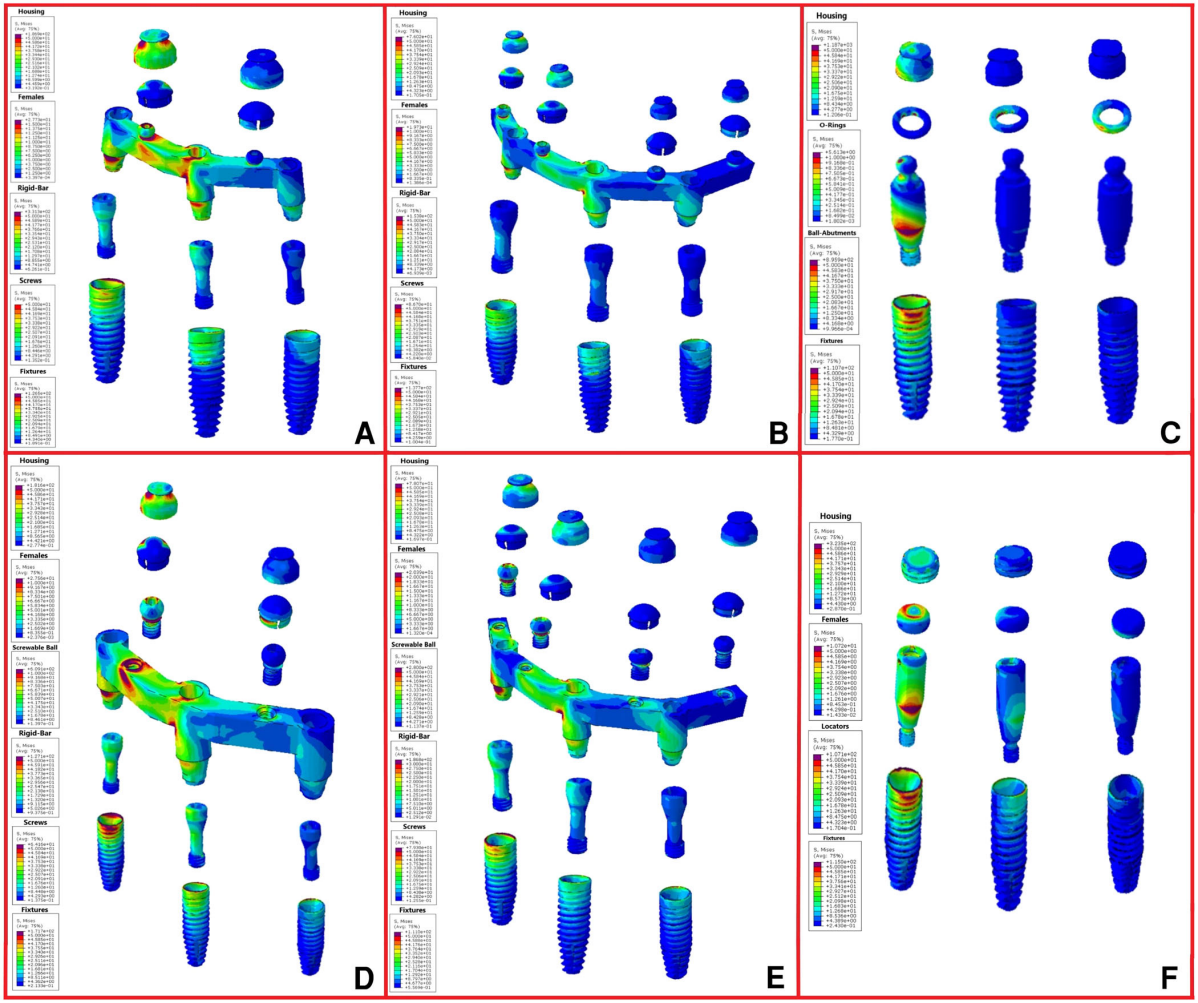
Stress distribution pattern under oblique loading in the six study groups: (A) WAX2, (B) WAX4, (C) Ball3, (D) CAD2, (E) CAD4, and (F) LOC3. The color‐coded indicators and their spatial distribution highlight the critical points and areas of higher stress concentration.

**Figure 7:**
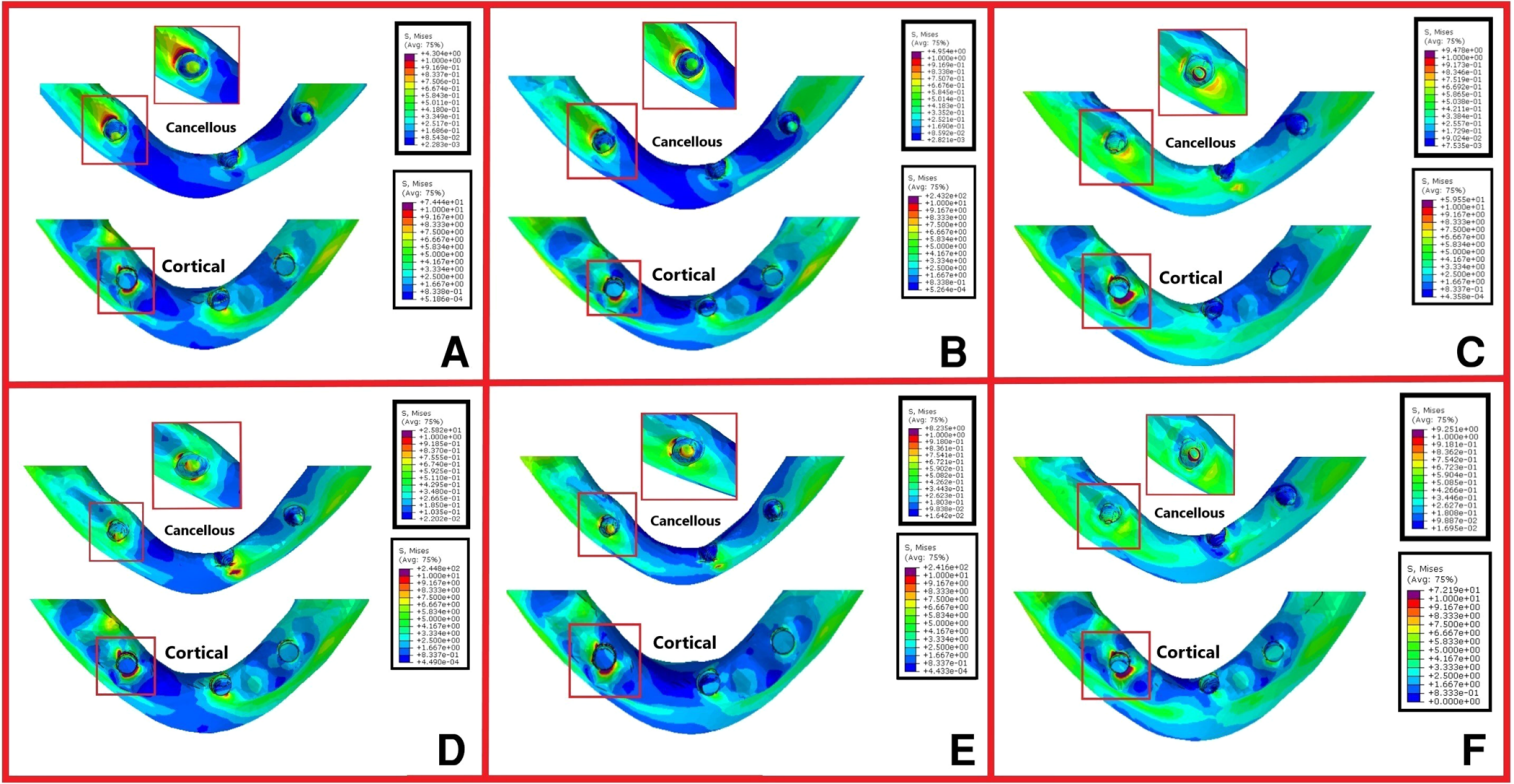
Stress distribution pattern of the cortical and cancellous bone in the six overdenture models under oblique loading: (A) WAX2, (B) WAX4, (C) Ball3, (D) CAD2, (E) CAD4, and (F) LOC3 in megapascals (MPa).

**Figure 8:**
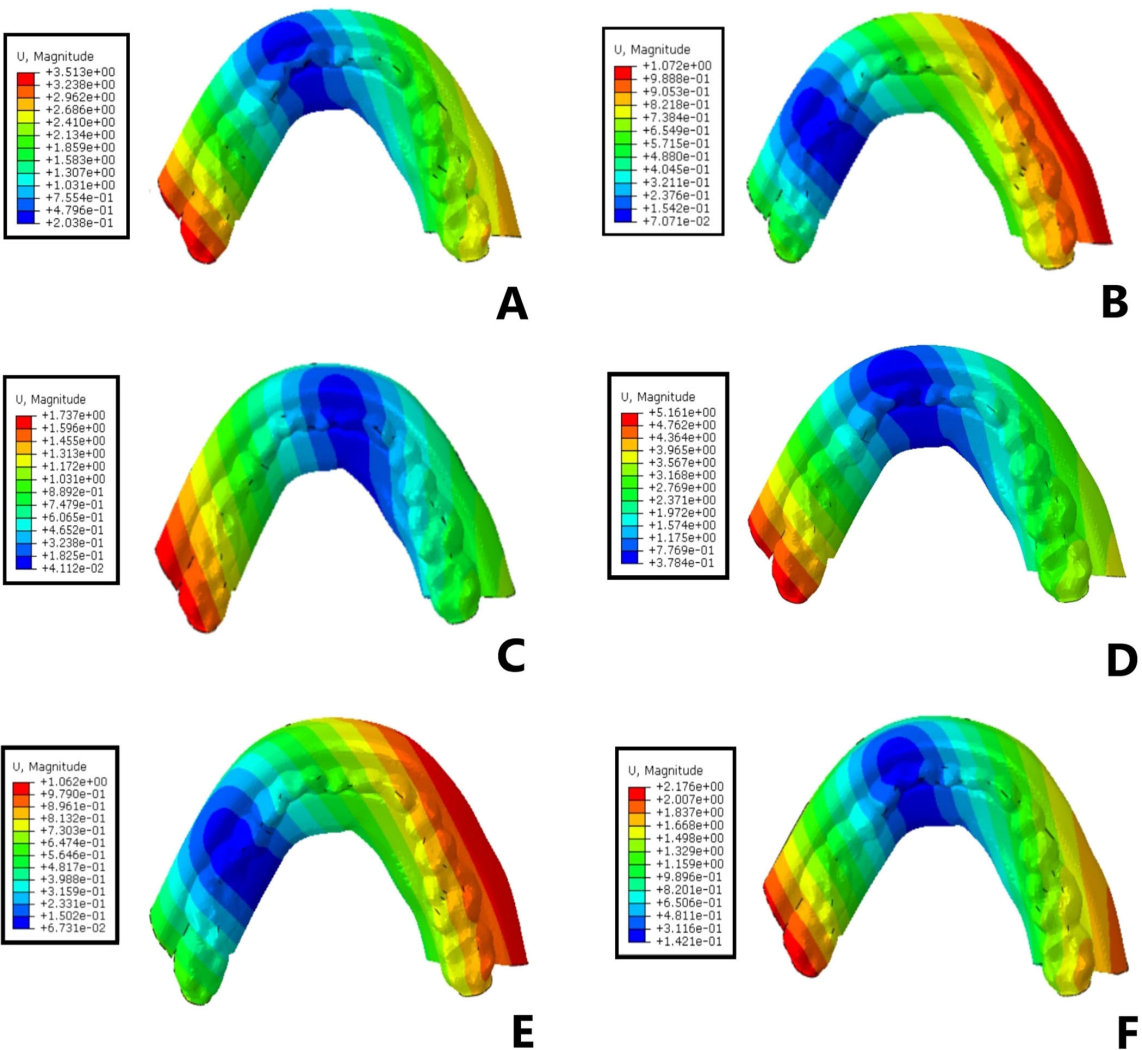
Denture displacement (mm) in the six overdenture models under oblique loading: (A) WAX2, (B) WAX4, (C) Ball3, (D) CAD2, (E) CAD4, and (F) LOC3.

The magnitudes of stress and displacement in the six study groups are shown by color codes in Figures 3, 4, 5 under vertical loading and in Figures 6, 7, 8 under oblique loading. In the attachments, the highest stress level under vertical and oblique loadings was seen in Ball3 (362 and 895 MPa) and the lowest stress level was recorded in lOC3 (50 and 107 MPa) and CAD2 (40 and 127 MPa). In implants, the highest stress level was recorded in WAX4 under vertical loading (262 MPa) and in CAD2 under oblique loading (170 MPa). The lowest stress level in implant was found in LOC3 (52 and 115 MPa under vertical and oblique loadings, respectively). The highest stress level in the cortical and cancellous bones was detected in CAD2 under oblique loading (244 and 26 MPa, respectively). The highest stress level in the cortical bone was found in WAX2 under vertical loading (34.4 MPa). Also, the highest stress level in the cancellous bone was found in Ball3 and LOC3 (602 and 506 MPa, respectively) under vertical loading. WAX4 showed the lowest stress level in the cancellous bone under vertical and oblique loadings (0.9 and 4.9 MPa, respectively). The highest stress level under vertical loading was found in two posterior (slightly more stress in the loading side) attachments, implants, screws, housing, the female part, and cortical and cancellous bones, except in the cantilever splinted attachment group (WAX4 and CAD4); also, anterior screw balls showed higher stress than posterior screw balls. LOC3 showed a more homogeneous stress distribution pattern. Under oblique loading, the highest stress level was found in the attachments, implants, screws, housing, the female part, and cortical and cancellous bones close to the loading point.

Under vertical loading, Balls3 showed the lowest displacement (0.74 mm). Other groups showed almost similar displacement. The highest displacement under oblique loading was observed in CAD2 (5.1 mm) and the lowest displacement was observed in CAD4 (1 mm).

## 4 Discussion

The null hypothesis of the study regarding no significant effect of attachment type or direction of loading on the magnitude of stress distribution in the overdenture and its adjacent structures was rejected. Oblique loading, compared with vertical loading, resulted in higher level of stress. Also, cortical bone had higher stress level than cancellous bone.

In splinted attachments, the difference in the stress distribution pattern may be due to the mechanical properties of different materials with the same design in CAD2 and WAX2, and also in the CAD4 and WAX4 groups. The highest stress level of stud attachments was concentrated at the connection (Ball3 and LOC3) and in the neck of the screw ball. Comparison of splinted and isolated designs of the four groups of WAX2, WAX4, CAD2, and CAD4 with Ball3 and LOC3 revealed lower stress distribution in the splinted models. As shown, the attachment near the loading force transfers the stress instead of tolerating it. The locator attachment showed the most homogeneous stress distribution pattern. Regarding the effect of attachment type, the results revealed the highest stress level in Ball3 and WAX2 and the lowest stress level in LOC3 and CAD2. The present results were in line with the findings of studies that compared the locator, ball, and splinted attachments, and reported the lowest stress level in the locator and the highest stress level in the ball attachment (Geramy and Habibzadeh 2018; Jiang et al. 2019). However, Barão et al. (2013) reported a lower stress level in the ball compared with the bar attachment. This difference can be due to differences in geometrical designs and the bar material (gold alloy). Shishesaz et al. (2016) reported that milled ball and bar attachments (with grade 4 titanium) had the highest level of stress and the conventional bar and ball attachments had the lowest level of stress. This finding may be due to differences in the geometrical designs of the milled ball and bar in their study, as balls were not screwed and were integrated with the bar structure. Accordingly, it may be concluded that a screw ball can serve as a stress breaker.

Under oblique loading, the isolated (ball and locator) groups showed a lower stress level in the cortical bone, whereas the cantilever (WAX4 and CAD4) designs caused lower stress distribution in bone under vertical loading. WAX2 showed a high stress level under vertical and oblique loadings, and CAD2 showed the lowest stress level in bone under vertical loading and the highest stress level under oblique loading. On using a waxed‐up bar, unlike the milled bars, a significant difference was noted in the stress distribution in bone when comparing WAX2 and WAX4 under vertical and oblique loadings. Assessment of the stress distribution pattern in the cancellous bone revealed higher stress levels in the isolated attachments, compared with the splinted types. Also, the waxed‐up bars yielded the lowest stress level in cancellous bone. CAD2 showed a different behavior under oblique (highest stress level) and vertical loadings, which was in contrast to the findings of Jiang et al. (2019). They demonstrated lower alveolar bone stress in the locator group than the bar group. However, they did not disclose the commercial brand of the dental implant and did not include implant threads in the meshed design. Kim and Hong (2016) demonstrated a more favorable stress distribution pattern on using the Hader bar compared with a milled bar. Shishesaz et al. (2016) demonstrated that using a bar attachment resulted in the lowest stress distribution in bone. Variations in the reported results may be attributed to differences in attachment designs by using a clip in the milled bar. Also, the balls on the milled bar were not screwed to it, and had been designed as one‐piece with the bar.

LOC3 showed the lowest stress level in dental implants. In splinted groups, a cantilever design (WAX4 and CAD4) increased the stress level in dental implants. The Ball3 group showed a different behavior under vertical (high stress level) and oblique (lowest stress level) loadings. The highest stress was recorded at the implant neck. These observations were in agreement with the results of other studies (Barão et al. 2013; Geramy and Habibzadeh 2018; Jiang et al. 2019; El‐Anwar et al. 2015). Kim and Hong (2016) reported higher stress levels in dental implants when using a milled bar, compared with the Hader bar, which may be due to different designs of the milled bar and the Hader bar.

In the assessment of stress distribution in the housings, the isolated group showed the highest level of stress (Ball3 > LOC3). In the splinted groups, those with four balls (with cantilever) showed lower stress level. Thus, it may be concluded that the type of material in milled and conventional bars plays a small role in this respect. Also, it may be concluded that splinting and increasing the number of balls and splinted attachments can leads to better stress distribution in housings. Consistent with the present results, Idzior‐Idzior‐Haufa et al. (2021) demonstrated that in the bar group, the CEKA attachment with a cantilever design resulted in lower stress level in the housing compared with no cantilever.

The lowest level of stress in the female part was found in LOC3. In the splinted groups, the presence of a cantilever (WAX4 and CAD4) decreased stress. The Ball3 group showed different behaviors under vertical and oblique (lowest stress level) loadings. It may be concluded that the surface area of the female part that is exposed to the force affects the stress level. The WAX2 group caused the lowest stress level in the screws. The cantilever groups (WAX4 and CAD4) caused higher stress accumulation in screws under oblique loading. Assessment of the stress distribution in the ball screwed to the milled bar revealed a lower stress level in the CAD2 group compared with CAD4. Finally, it may be concluded that in the splinted group, the cantilever design (WAX4 and CAD4) and increasing the number of balls decreased the stress level in the matrix components (housing and female part) and the screw ball.

The lowest displacement was observed in the splinted groups with a cantilever (WAX4 and CAD4) under oblique loading, whereas the splinted groups without a cantilever (WAX2 and CAD2) had the highest displacement. Also, Idzior‐Idzior‐Haufa et al. (2021) reported that the bar group with the CEKA attachment and a cantilever design had less displacement than the bar without a cantilever. Moreover, they concluded that higher rigidity of the prosthetic components decreased displacement. This finding was in line with the present results in the WAX2 and CAD2 models; however, no significant difference was found between the WAX4 and CAD4 models. Thus, it may be concluded that the attachment design is more effective than the material type in milled and conventional bars on displacement. Under vertical loading, the isolated attachment (particularly Ball3) had the least displacement. This result may be due to differences in the female part (ring vs. cap). However, the milled bars had different behaviors under vertical loading and showed greater displacement.

Few studies have evaluated overdenture supported by three dental implants clinically; however, Emami et al. (2019), who carried out a clinical trial study, reported that the addition of a midline to an existing mandibular two‐implant overdenture resulted in a decrease in the anteroposterior movement, stability of the overdenture, and ability to speak significantly. Also, Celebic et al. (2023), who carried out a 5‐year randomized clinical trial, reported that in patients with narrow mandibular ridges, the insertion of three implant for overdenture retention can be equally the insertion of four implant.

This study is among the few to compare different attachment types by precise evaluation of stress distribution patterns in all prosthetic components, dental implants, and bone in an overdenture supported by three dental implants. The major limitation of this study was the inherent limitation that is present in finite element studies. Assessment of only one type of overdenture supported by three dental implants was another limitation of this study. Further studies are recommended to assess the stress distribution pattern in different designs of conventional bars in comparison with different designs of milled bars. Also, these parameters should be evaluated in overdentures supported by different numbers of implants in vitro, and also in the clinical setting.

## 5 Conclusion

Considering the limitations of this study, the results showed that:

1. The ball attachment caused the highest stress level in the attachment, housing, and female part compared with other attachments.
2. The bars without a cantilever (CAD2 and WAX2) showed the highest displacement.
3. The bar and ball without a cantilever may need greater care for replacement of the female part.
4. Comparison of CAD2 and CAD4 revealed the superiority of CAD4 in terms of the stress distribution pattern and displacement. In general, CAD4 showed acceptable results in the splinted group. Also, the cantilever design was more acceptable in the milled bar compared with the cast bar.
5. LOC3 showed a more homogeneous and more favorable stress distribution pattern in prosthetic components, especially under oblique loading.

## Author Contributions

**Negin Aminianpour:** investigation, visualization, writing–original draft preparation, writing–review. **Marzieh Alikhasi:** methodology, writing–review and editing, supervision. **Mostafa Shabanpour Kasari:** model designing and meshing, writing–review. **Hashem Yousefi:** finet element analysis and writing–review. **Hakimeh Siadat:** project administration, conceptualization, methodology, writing–review and editing.

## Ethics Statement

As this was an in vitro study, there was no need for ethics approval.

## Conflicts of Interest

The authors declare no conflicts of interest.

## Data Availability

The study data are available from the corresponding author upon reasonable request.
